# Significance of Convection and Internal Heat Generation on the Thermal Distribution of a Porous Dovetail Fin with Radiative Heat Transfer by Spectral Collocation Method

**DOI:** 10.3390/mi13081336

**Published:** 2022-08-17

**Authors:** G. Sowmya, Maha M. A. Lashin, M. Ijaz Khan, R. S. Varun Kumar, K. C. Jagadeesha, B. C. Prasannakumara, Kamel Guedri, Omar T Bafakeeh, El Sayed Mohamed Tag-ElDin, Ahmed M. Galal

**Affiliations:** 1 Department of Mathematics, M S Ramaiah Institute of Technology, Bangalore 560054, India; 2 Electrical Engineering Department, Princess Nourah bint Abdulrahman University, Riyadh 11564, Saudi Arabia; 3 Department of Mathematics and Statistics, Riphah International University I-14, Islamabad 44000, Pakistan; 4 Department of Studies in Mathematics, Davangere University, Tholhunse 577002, India; 5 Department of Mathematics, I.D.S.G. Government First Grade College, Chikkamagaluru 577101, India; 6 Mechanical Engineering Department, College of Engineering and Islamic Architecture, Umm Al-Qura University, P.O. Box 5555, Makkah 21955, Saudi Arabia; 7 Department of Industrial Engineering, Jazan University, Jazan 82822, Saudi Arabia; 8 Faculty of Engineering and Technology, Future University in Egypt, New Cairo 11835, Egypt; 9 Mechanical Engineering Department, College of Engineering, Prince Sattam Bin Abdulaziz University, Wadi ad-Dawaser 11991, Saudi Arabia; 10 Production Engineering and Mechanical Design Department, Faculty of Engineering, Mansoura University, Mansoura 35516, Egypt

**Keywords:** extended surface, porous extended surface, dovetail fin, spectral collocation method (SCM)

## Abstract

A variety of methodologies have been used to explore heat transport enhancement, and the fin approach to inspect heat transfer characteristics is one such effective method. In a broad range of industrial applications, including heat exchangers and microchannel heat sinks, fins are often employed to improve heat transfer. Encouraged by this feature, the present research is concerned with the temperature distribution caused by convective and radiative mechanisms in an internal heat-generating porous longitudinal dovetail fin (DF). The Darcy formulation is considered for analyzing the velocity of the fluid passing through the fin, and the Rosseland approximation determines the radiation heat flux. The heat transfer problem of an inverted trapezoidal (dovetail) fin is governed by a second-order ordinary differential equation (ODE), and to simplify it to a dimensionless form, nondimensional terms are utilized. The generated ODE is numerically solved using the spectral collocation method (SCM) via a local linearization approach. The effect of different physical attributes on the dimensionless thermal field and heat flux is graphically illustrated. As a result, the temperature in the dovetail fin transmits in a decreasing manner for growing values of the porosity parameter. For elevated values of heat generation and the radiation-conduction parameter, the thermal profile of the fin displays increasing behavior, whereas an increment in the convection-conduction parameter downsizes the thermal dispersal. It is found that the SCM technique is very effective and more conveniently handles the nonlinear heat transfer equation. Furthermore, the temperature field results from the SCM-based solution are in very close accordance with the outcomes published in the literature.

## 1. Introduction

Fins or extended surfaces are an efficient method to strengthen the dissipation of heat from a hot surface. They are utilized in several industrial applications, including processing plants, aerospace, and electronics. It is well recognized that the operations of certain industrial sectors, such as chemical, nuclear, and power plants, considered the increased production of an enormous amount of heat, necessitating a meticulous technique of heat dissipation. Even though diverse refrigerating strategies have been used to disperse heat from thermal systems, fins serve a crucial role in increasing the rate of heat transmission. It is especially possible to speed up heat transmission between a solid and an adjacent liquid by using an expanded surface. The analysis of fin design has attracted a lot of attention in past years, and much importance has been placed on the innovation of apposite techniques for solving nonlinear fin issues. Unsteady thermal dispersal in a fin with different cross-section profiles, namely, rectangular, concave, triangular, and convex, was discussed by Mosayebidorcheh et al. [1]. Moreover, they considered internal heat production and thermal conductivity as linear thermal components, whereas the convective heat coefficient is regarded as a nonlinear thermal function. Turkyilmazoglu [2] explored the heat transfer and thermal dispersal through a fin of exponential profile. Gouran et al. [3] debriefed the thermal variations in a fin of a rectangular profile by considering the conductive and convective heat flux with internal heat production. The features of stress caused due to the thermal load on an annular fin were explained by Kumar et al. [4] with the consideration of thermal dispersion driven by convection and heat generation. The impact of convection and a magnetic environment was considered by Sowmya et al. [5] to explicate the thermal features of a rectangular-profiled annular fin.

Heat transport mechanisms in porous media are important in a variety of applications, including chemical catalytic reactors, geothermal energy extraction, thermal insulation, heat exchanger design, and oil exploration. As a result, they have gained considerable priority in recent years of research [6,7,8,9,10]. Porous fins designed with a porous material are more effective and compact in heat exchange compared to all other fins in many industrial applications such as heat exchangers and solar collectors. Even though porous fins have limited thermal conductivity, they significantly strengthen fluid flow blending and enhance the contact between the material’s surface area and the cooling liquid inside, and the use of porous materials has been noticed as an efficient and appropriate procedure of heat transmission enrichment. Consequently, the mechanism of heat transport from porous fins has evoked interest in many investigators. Das and Kundu [11] presented a significant approach for enhancing the rate of energy transmission through the porous fin by applying the combined magnetic and electrical effects. The collocation method with sine transformation was implemented by Nabati et al. [12] to explore the numerical result for the thermal equation of a permeable fin. Wang et al. [13] analyzed the upshot of inclination angle on the thermal diffusion of a radiative permeable fin with convection and internal heat generation. The nature of the thermal field of a longitudinal fin having various cross-section profiles was scrutinized by Hosseinzadeh et al. [14] with the consideration of wet surface conditions. Production of entropy in the permeable fin of an exponential profile was expounded by Din et al. [15] with radiation, internal heat production, and convective mechanisms. Recently, Kumar et al. [16] considered the porous concave fin with an inclined position to examine the unsteady thermal distribution in the extended surface. The rectangular fin has been explored extensively owing to its low cost and ease of fabrication. Fins with a parabolic structure, on the other hand, are usually recommended over rectangle profile fins in design features where weight is a consideration, such as in aircraft applications. However, due to their curved configuration, parabolic fins are more challenging to fabricate and thereby more expensive. Furthermore, if an effort is made to fabricate a triangular fin, the sharp edge causes safety concerns. A convenient and more generalizable effect is caused by a trapezoidal fin rather than by other shaped fins. As a result, in terms of light weight and safety, a trapezoidal fin is one of the best alternatives. Thus, fins with a constant slope profile, also known as trapezoidal profiles, are utilized in numerous applications, and several researchers considered trapezoidal-profiled fins in their thermal analysis [17,18,19,20]. Recently, Gireesha et al. [21] examined the temperature variation and heat transmission in an inclined permeable straight fin of dovetail and trapezoidal profile with radiation and convective heat transfer.

The spectral collocation method (SCM) solves nonlinear differential equations faster and more effectively. It is the methodological approach to approximation in which residuals (or errors) are strictly controlled. This methodology’s prominence arises from its superior characteristics to traditional finite difference approaches, and they can perform quicker convergent approximations. The simultaneous verification of a solution’s spectrum and verifying of resolution is enabled by the dual illustration in physical and transformed space. Particular symmetries in a solution can be manipulated using spectral methods. As an outcome, inconsistencies in phase and dissipation in the procedure are minor or nonexistent. The primary benefit of these methodologies is their consistency for a specific number of unknowns. Finite-difference and finite-element techniques produce only algebraic convergence rates. On the other hand, SCM provides an exponential rate of convergence for smooth problems in simple models. The most prevalent spectral representations are Galerkin, collocation, and tau techniques. Due to its computational simplification and good accuracy, the SCM has been shown to be an important strategy in the engineering and science fields [22,23,24]. In previous years, the SCM was employed to attain the solution for the heat transfer equation of fins, rods, moving fins, and fins with irregular cross-section areas [25,26,27].

Upon performing a detailed literature survey, it was observed that Gireesha et al. [21] and Kang [28] scrutinized temperature distribution in a reversed trapezoidal (dovetail) fin. Dovetail fins can be employed in double pipe heat exchangers, which transfer more heat with the same base area as a rectangle-structured fin. By using the dovetail fin, a better heat dispersal over the extended area furnished by the reversed trapezoidal can be acquired. However, this type of fin has received less attention. On the other side, the use of microchannel heat sinks with fin arrangements has received a great deal of attention from authors [29,30]. An increased surface area aided by improved blending is the core benefit of the fin configuration in the microchannel. In addition, considerable effort has been devoted to improving convective heat transfer and thus reducing thermal resistance by fully or partially implanting porous fins, blocks, or baffles into the microchannel. Therefore, the present investigation elucidates the thermal distribution and transfer of heat through a porous dovetail fin with convection and radiation mechanisms. Moreover, the novelty of this investigation is to scrutinize the effect of convection, internal heat generation, and radiation on thermal distribution through a porous dovetail fin. The Darcy formulation is considered for analyzing the fluid velocity passing via the fin, and the radiation heat flux is governed by the Rosseland approximation. The ODE portraying the heat transport of the porous fin is nondimensionalized and numerically simplified using SCM. Graphs and tables are developed to observe the characteristics of the thermal profile and temperature gradient for various values of dimensionless parameters.

## 2. Mathematical Formulation

**Figure 1:**
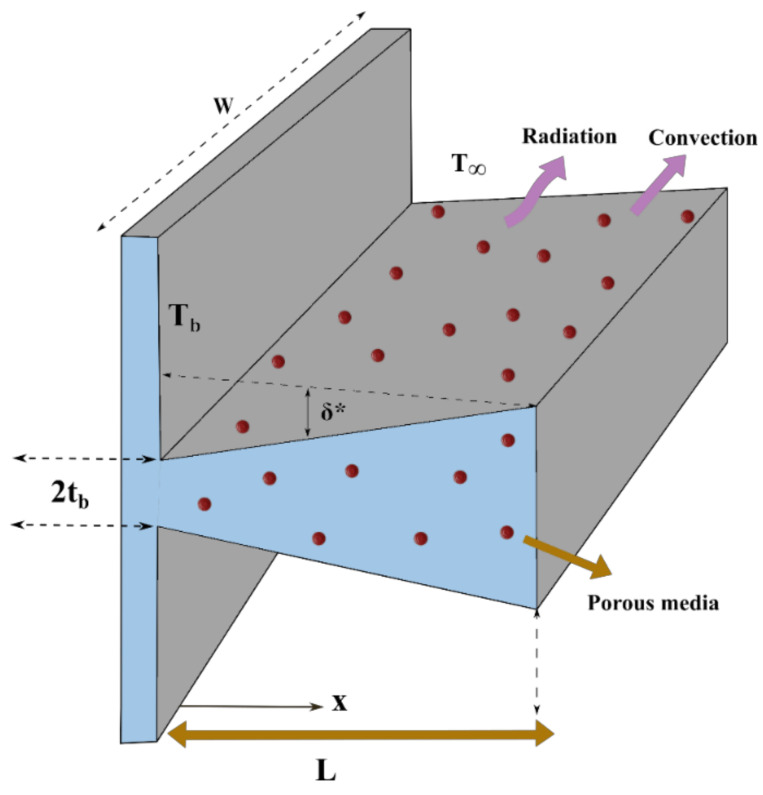
Physical model of a dovetail fin.

The steady-state heat passage of a porous DF with internal heat generation is conferred in this model, as displayed in Figure 1. Heat is dissipated from the fin’s surface to the ambient environment at temperature $T_{\infty }$ via convection and radiation. The permeable medium is assumed to be saturated with a single-phase, homogeneous, and isotropic liquid. Heat exchange occurs in a permeable fin due to heat conduction and thermal radiation transfer heat from the adjacent side. Transfer of heat energy is due to natural convection that occurs when the fluid’s mass flows through the porous structure.

The mathematical model for the present fin problem is mentioned as follows (see Hatami and Ganji [31], Kundu and Yook [32]):

(1) $$q_{x}-q_{x+dx}=\left(1-\phi \right)h^{*}w\left(T-T_{\infty }\right)dx+\dot{m}c_{p}\left(T-T_{\infty }\right)+w\varepsilon^{*}\sigma \left(T^{4}-T_{\infty }^{4}\right)dx-w\;t(x)\left(1-\phi \right)q_{\mathrm{int}}^{*}\left(T\right)$$

At the fin’s base, the heat flux vector of combined radiation and conduction is prescribed as

(2) $$q_{b}=q_{cond}+q_{rad}$$

and the term for heat conductivity may be represented as follows using Fourier’s rule of conduction (see Rostamiyan et al. [33]):

(3) $$q_{cond}=-k_{eff}A_{cr}\frac{dT}{dx}$$

where, $k_{eff}=\left(1-\phi \right)k_{s}+\phi k_{f}$ is the effectual thermal conductivity and

(4) $$A_{cr}=w\;t\left(x\right),$$

is the area of cross-section.

The thermal radiative flux term is represented using the Rosseland diffusion approximation as (see Bhanja et al. [34] and Alhejaili et al. [35])

(5) $$q_{rad}=-\frac{4\sigma A_{cr}}{3\beta_{R}^{*}}\frac{dT^{4}}{dx}$$

Now, Equation (1) can be written as

(6) $$\begin{array}{l} -\frac{dq_{b}}{dx}-\left(1-\phi \right)wh^{*}\left(T-T_{\infty }\right)+w\;t(x)\left(1-\phi \right)q_{\mathrm{int}}^{*}\left(T\right)-\dot{m}c_{p}\left(T-T_{\infty }\right)\\ \;\;\;\;\;\;\;\;\;\;\;\;\;\;\;-w\varepsilon^{*}\sigma \left(T^{4}-T_{\infty }^{4}\right)=0 \end{array}$$

and $\dot{m}$ is demarcated as

(7) $$\dot{m}=\rho_{f}\upsilon_{w}\left(x\right)w\phi \;dx$$

where the passage velocity $\upsilon_{w}\left(x\right)$ is represented as follows by considering Darcy’s model (see Moradi et al. [36] and Weera et al. [37]):

(8) $$\upsilon_{w}\left(x\right)=\frac{gK\beta_{f}}{\nu_{f}}\left(T-T_{\infty }\right)$$

Utilizing Equations (3)–(5), Equation (6) is symbolized as follows:

(9) $$\begin{array}{l} \frac{d}{dx}\left[k_{eff}w\;t(x)\frac{dT}{dx}+\frac{4\sigma w\;t(x)}{3\beta_{R}^{*}}\frac{dT^{4}}{dx}\right]-\left(1-\phi \right)wh^{*}\left(T-T_{\infty }\right)+w\;t(x)\left(1-\phi \right)q_{\mathrm{int}}^{*}\left(T\right)\\ \;\;\;\;\;\;\;\;\;\;\;\;\;\;\;-\dot{m}c_{p}\left(T-T_{\infty }\right)-w\varepsilon^{*}\sigma \left(T^{4}-T_{\infty }^{4}\right)=0 \end{array}$$

From the perspective of energy transfer, the porous fin may be referred to as the simple model when it is only operated in a natural convection environment. To formulate such a kind of heat transfer equation, the approach depends only on Darcy’s model and energy balance. However, energy dissipation from the permeable fin is dependent not only on natural convection but also on solid surface convection and radiative heat exchange with the adjacent environment. Therefore, the scenario analyzed in this investigation is when a slight temperature variance occurs inside the material while the heat flows. Moreover, it has been revealed that the term $T^{4}$ can be demonstrated as a linear thermal function in such a particular circumstance (see Kiwan [38] and Madhura et al. [39]),

(10) $$i.e.,\;T^{4}≅4T_{\infty }^{3}T-3T_{\infty }^{4}$$

Using Equation (10) in Equation (9) yields

(11) $$\begin{array}{l} \frac{d}{dx}\left[\left(\left(1-\phi \right)k_{s}+\phi k_{f}\right)\;\frac{dT}{dx}+\phi \frac{16\sigma T_{\infty }^{3}}{3\beta_{R}^{*}}\frac{dT}{dx}\right]-\left(1-\phi \right)h^{*}\left(T\right)\left(T-T_{\infty }\right)\\ +\left(1-\phi \right)q_{\mathrm{int}}^{*}\left(T\right)-\phi \frac{\rho_{f}c_{p}gK\beta_{f}}{\nu_{f}}{\left(T-T_{\infty }\right)}^{2}-\varepsilon^{*}\sigma \left(T^{4}-T_{\infty }^{4}\right)=0 \end{array}$$

The local semi-fin thickness is specified as

(12) $$t(x)=t_{b}-\delta^{*}\left(\frac{x}{L}\right)$$

Putting Equation (12) into Equation (11) leads to

(13) $$\begin{array}{l} \frac{d}{dx}\left[\left(\left(1-\phi \right)k_{s}+\phi k_{f}\right)\left(t_{b}-\delta^{*}\left(\frac{x}{L}\right)\right)\;\frac{dT}{dx}+\phi \left(t_{b}-\delta^{*}\left(\frac{x}{L}\right)\right)\frac{16\sigma T_{\infty }^{3}}{3\beta_{R}^{*}}\frac{dT}{dx}\right]-\left(1-\phi \right)h^{*}\left(T\right)\left(T-T_{\infty }\right)-\phi \frac{\rho_{f}c_{p}gK\beta_{f}}{\nu_{f}}{\left(T-T_{\infty }\right)}^{2}-\\ \varepsilon^{*}\sigma \left(T^{4}-T_{\infty }^{4}\right)+\left(1-\phi \right)\left(t_{b}-\delta^{*}\left(\frac{x}{L}\right)\right)q_{\mathrm{int}}^{*}\left(T\right)=0 \end{array}$$

$h^{*}\left(T\right)$ is generally assumed to be constant in the traditional analysis of fin problems. However, when the fin is being cooled by a boiling two-phase flow, where $h^{*}\left(T\right)$ is highly predicated on the local temperature surplus, such an assumption could lead to significant error. Thus, $h^{*}\left(T\right)$ is supposed to be a nonlinear component of the local temperature difference T causing the convection process and is given as follows (see Sowmya et al. [40]):

(14) $$h^{*}\left(T\right)=h_{b}{\left[\frac{T-T_{\infty }}{T_{b}-T_{\infty }}\right]}^{n}$$

where n is a dimensionless constant that can range between −6.6 and 5, or, in the majority of practical uses, between −3 and 3.

In addition,

(15) $$q_{\mathrm{int}}^{*}\left(T\right)=q_{\infty }^{*}\left[1+\lambda \left(T-T_{\infty }\right)\right]$$

The boundary conditions for DF are

(16) $$T\left(0\right)=T_{b},\;{\frac{dT}{dx}}_{x=L}=0$$

Nondimensionalization is executed utilizing the following nondimensional terms:

(17) $$\Theta =\frac{T-T_{\infty }}{T_{b}-T_{\infty }},\;\mathrm{and}X=\frac{x}{L}$$

and Equation (13) can be simplified as

(18) $$\begin{array}{l} \frac{d}{dX}\left[\left(\left(1-\phi \right)+\phi k_{r}\right)\left(1-CX\right)\frac{d\Theta }{dX}+4\;R_{d}^{*}\;\phi \left(1-CX\right)\frac{d\Theta }{dX}\right]-\left(1-\phi \right)Nc\;\Theta^{n+1}-\phi S_{H}\Theta^{2}-Nr\left[{\left(Nt+\Theta \right)}^{4}-Nt^{4}\right]+\\ \left(1-\phi \right)\left(1-CX\right)Q_{\mathrm{int}}\left(1+\gamma \;\Theta \right)=0 \end{array}$$

Nondimensional parameters in the above equation are listed in Table 1, and C is supposed to be negative $\left(C<0\right)$, related to DF (Gireesha et al. [21]).

Correspondingly, Equation (16) is reduced to

(19) $$\Theta \left(0\right)=1,\;{\left.\frac{d\Theta }{dX}\right|}_{X=1}=0$$

Now, Equation (18) is rearranged as follows to reduce nonlinear effects:

(20) $$\begin{array}{l} \frac{d}{dX}\left[\left(\left(1-\phi \right)+\phi k_{r}\right)\left(1-CX\right)\frac{d\Theta }{dX}+4\;R_{d}^{*}\;\phi \left(1-CX\right)\frac{d\Theta }{dX}\right]+\chi_{1}Nc\;\Theta^{m}+\chi_{2}Nr\;\Theta^{4}=\\ \left(1-\phi \right)Nc\;\Theta^{n+1}+\phi S_{H}\Theta^{2}+Nr\left[{\left(Nt+\Theta \right)}^{4}-Nt^{4}\right]-\left(1-\phi \right)\left(1-CX\right)Q_{\mathrm{int}}\left(1+\gamma \;\Theta \right)+\chi_{1}Nc\;\Theta^{m}+\chi_{2}Nr\;\Theta^{4} \end{array}$$

**Table 1:** Nondimensional parameters present in the dimensionless fin equation.

Definition	Nondimensional Parameters
$Nc=\frac{h_{b}L^{2}}{k_{s}t_{b}}$	Convective–conductive parameter
$Nr=\frac{\;\sigma \;\varepsilon^{*}L^{2}}{k_{s}t_{b}}{\left(T_{b}-T_{\infty }\right)}^{3}$	Radiation number
$C=\frac{\delta^{*}}{t_{b}}$	Fin taper ratio
$\gamma =\lambda \left(T_{b}-T_{\infty }\right)$	Heat generation parameter
$S_{H}=\frac{\rho_{f}c_{p}gK\beta_{f}L^{2}}{\nu_{f}k_{s}t_{b}}\left(T_{b}-T_{\infty }\right)$	Porosity parameter
$k_{r}=\frac{k_{f}}{k_{s}}$	Relative thermal conductivity
$Q_{\mathrm{int}}=\frac{q_{\infty }^{*}\;L^{2}}{\left(T_{b}-T_{\infty }\right)k_{s}}$	Heat generation number
$R_{d}^{*}=\frac{4\sigma }{3\beta_{R}^{*}k_{s}}{\left(T_{\infty }\right)}^{3}$	Radiation–conduction parameter
$Nt=\frac{T_{\infty }}{T_{b}-T_{\infty }}$	Temperature ratio parameter

## 3. Spectral Collocation Method Formulation

Over the past few decades, a significant attempt has been made to find exact solutions for mathematical models indicating the physical phenomenon. However, various models have been explored numerically and analytically by some investigators [41,42,43]. In the present work, SCM with Chebyshev–Gauss–Lobatto collocation points (Khorrami [44], Sun and Li [45]) are utilized for spatial discretization of Equation (18).

(21) $$\xi_{i}=-\cos\left[\frac{\pi \left(j-1\right)}{Ν-1}\right],\;j=1,2\ldots ,Ν$$

The succeeding transformation maps $\left[X_{\min},\;X_{\max}\right]$ into [−1,1] to meet the Chebyshev polynomial requirement.

(22) $$2X=\left[\left(X_{\max}-X_{\min}\right)\xi +\left(X_{\max}+X_{\min}\right)\right]$$

As per the SCM principle, Θ on collocation points can be simulated by Lagrange interpolation polynomials (see Khater et al. [22], Ma and Huang [23]).

(23) $$\Theta \left(\xi \right)\approx \sum_{j=1}^{Ν}\Theta \left(\xi_{j}\right)g_{j}\left(\xi \right)$$

where

(24) $$g_{j}\left(\xi \right)=\frac{w_{j}^{\prime }/\left(\xi -\xi_{j}\right)}{\sum_{l=1}^{Ν}w_{l}^{\prime }/\left(\xi -\xi_{j}\right)}$$

and

(25) $$\begin{array}{l} w_{l}^{\prime }={\left(-1\right)}^{l-1}\Delta_{l}^{\prime },\\ \Delta_{l}^{\prime }=\left\{\begin{array}{l} 1/2,\;\;l=1,Ν\\ 1,\;\;\;\;\mathrm{otherwise} \end{array}\right. \end{array}$$

Putting Equation (21) into Equation (20) yields the matrix structure of a spectral discretized algebraic expression.

(26) $$\sum_{l=1}^{Ν}{Ε}_{j,l}\Theta_{l}=\Gamma_{j},\;\;\;j=1,2\ldots Ν$$

where the matrices element expressions ${Ε}_{j,l}$ and $\Gamma_{j}$ are demarcated as

(27) $$E_{j,l}=\left\{\begin{array}{c} -\left(k_{r}\phi -\phi +1\right)C\left(\frac{2}{X_{\mathrm{mix}}-X_{\min}}\right)D_{jl}^{\left(1\right)}+\left(k_{r}\phi -\phi +1\right)\left(-C\left(\frac{X_{\max}-X_{\min}}{2}\right)+1\right){\left(\frac{2}{X_{\max}-X_{\min}}\right)}^{2}D_{jl}^{\left(2\right)}-\\ 4R_{d}^{*}\phi C\left(\frac{2}{X_{\max}-X_{\min}}\right)D_{jl}^{\left(1\right)}+4R_{d}^{*}\phi \left(-C\left(\frac{X_{\max}-X_{\min}}{2}\right)+1\right)D_{jl}^{\left(2\right)}+\\ \chi_{1}Nc{\left(\Theta_{j}^{*}\right)}^{m}+\chi_{2}Nr{\left(\Theta_{j}^{*}\right)}^{4}\;\;\;\;\;\;\;\;\;\;\;\;\;\;\;\;\;\;\;\;\;,j=l\\ \left(k_{r}\phi -\phi +1\right)\left(-C\left(\frac{X_{\max}-X_{\min}}{2}\right)+1\right){\left(\frac{2}{X_{\max}-X_{\min}}\right)}^{2}D_{jl}^{\left(2\right)}-4R_{d}^{*}\phi C\left(\frac{2}{X_{\max}-X_{\min}}\right)D_{jl}^{\left(1\right)}\\ +4R_{d}^{*}\phi \left(-C\left(\frac{X_{\max}-X_{\min}}{2}\right)+1\right)D_{jl}^{\left(2\right)}-\left(k_{r}\phi -\phi +1\right)C\left(\frac{2}{X_{\max}-X_{\min}}\right)D_{jl}^{\left(1\right)}\;\;\;\;\;\;\;\;\;,j\neq l \end{array}\right.$$

and

(28) $$\begin{array}{l} \Gamma_{j}=\left(1-\phi \right)Nc{\left(\Theta_{j}^{*}\right)}^{n+1}+\phi S_{H}{\left(\Theta_{j}^{*}\right)}^{2}+Nr\left({\left(Nt+\Theta_{j}^{*}\right)}^{4}-Nt^{4}\right)-\left(1-\phi \right)\left(1-C\left(\frac{X_{\max}-X_{\min}}{2}\right)\right)Q_{\mathrm{int}}\left(1+\gamma \;\left(\Theta_{j}^{*}\right)\right)+\\ \;\;\;\;\;\chi_{1}Nc\;\Theta^{m}+\chi_{2}Nr\;\Theta^{4} \end{array}$$

Additionally, the spectral discretization of boundary conditions is provided in Ma et al. [26].

## 4. Results and Discussions

This segment explores the consequence of several parameters in the thermal analysis of a porous DF. The SCM technique is utilized to solve the nondimensional energy transfer issue. In addition, graphs and tables are used to examine the behavior of temperature profile $\Theta \left(X\right)$ and heat flux $-\Theta^{\prime }\left(0\right)$ with the consequence of various thermal parameters, including convection–conduction parameter $\left(1\leq Nc\leq 5\right)$, radiation–conduction parameter $\left(0.5\leq R_{d}^{*}\leq 5\right)$, temperature ratio parameter $\left(0.1\leq Nt\leq 0.5\right)$, porosity parameter $\left(0\leq S_{H}\leq 20\right)$, radiation number $\left(1\leq Nr\leq 10\right)$, coefficients of fin taper ratio $\left(-0.1\leq C\leq -0.3\right)$, and heat generation number $\left(0\leq Q_{\mathrm{int}}\leq 1\right)$. Table 2 portrays the variation in thermal profile and heat flux for varying values of porous fraction ϕ (0 and 0.1), thermal conductivity parameter $k_{r}$ (0 and 0.3), Nc (2 and 5), $R_{d}^{*}$ (0.5 and 0.7), temperature ratio parameter Nt (0.1 and 0.3), porosity parameter $S_{H}$ (0 and 7), and radiation number Nr (2 and 4), while the coefficients of fin taper ratio C=−0.3, heat generation number $Q_{\mathrm{int}}=0.8$, power index n=0, and fin length are taken as constant.

The values of thermal profile of the porous fin are noticed to be diminished as the ϕ, Nc, Nr, Nt, and $S_{H}$ increases, but the heat flux augments for enhancement in these parameter values. Elevated scales of $k_{r}$ and $R_{d}^{*}$ lead to the enhancement of the temperature profile and cause the decrement of heat flux. For the various magnitude of selected parameters, Table 3 was constructed to compare the present results (PR) of heat flux to the benchmark solution of Gorla and Bakier [46] (GB).

**Figure 2:**
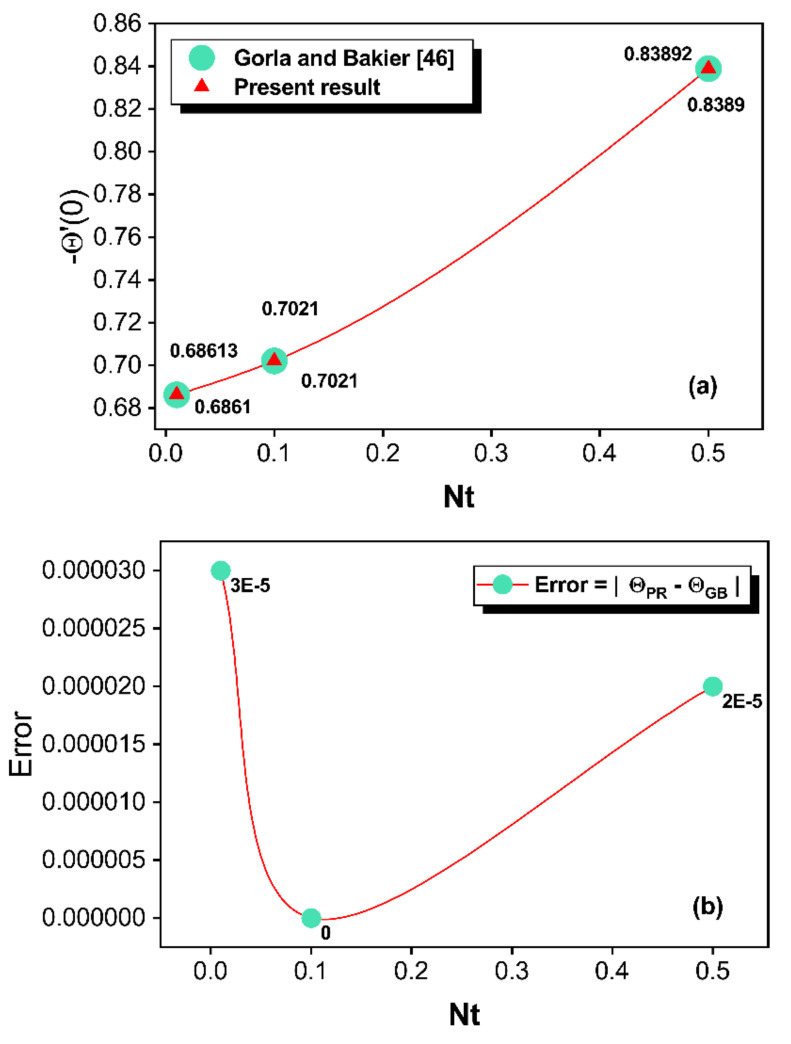
(**a**,**b**) Comparison of the results and error estimations.

The comparison of the current analysis to the available result exhibits an excellent agreement with a maximum error of 0.00003. This analysis can be studied in detail by considering the graphical structure. Figure 2a indicates a comparison of the current numerical scheme with that of Gorla and Bakier [46] by taking $C=Nc=n=Q_{\mathrm{int}}=\gamma =R_{d}^{*}=0$, ϕ=1, and $k_{r}=1$. In this figure, the value of $-\Theta^{\prime }\left(0\right)$ is compared by setting the $S_{H}$ and Nr values to 1 and 0.1, respectively, and $-\Theta^{\prime }\left(0\right)$ also depends on the varying values of $Nt\left(0.01,\;0.1,\;0.5\right)$. From Figure 2a, an appropriate consistency between the data from this analysis and those published in Ref. [46] is observed, implying that the numerical technique for the simulation is accurate. Observations indicate an improvement in heat flux as a result of an increment in Nt, which is the ratio of ambient temperature to the difference among the base and ambient temperatures. The physical justification is that greater emission of heat into the environment is correlated with higher scales of Nt, which causes the thermal distribution to decrease and heat flux to augment. Figure 2b is provided to estimate the error between these comparisons using the correlation $Error=\left|\Theta_{PR}-\Theta_{GB}\right|$. According to the comparative outcomes, the maximum deviances are identified for Nt=0.01 (see Figure 2b). Furthermore, the comparisons and error estimations show that the SCM has good accuracy due to their exponential convergence.

**Figure 3:**
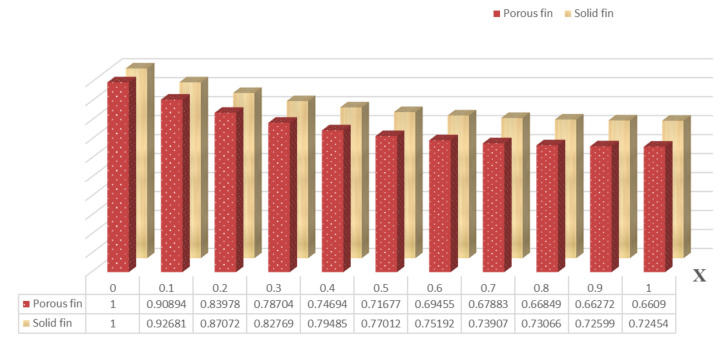
Thermal distribution along the longitudinal direction.

The primary difference between solid and porous fins is the presence of an extensive number of pores involved with the permeable fin, which permits liquid to flow via these holes. Based on the current examination, Figure 3 is designed to demonstrate the thermal distribution and heat transfer rate in porous and solid dovetail fins as a component of fin length. At X=0, the thermal profile for both solid and porous dovetail fins attain their maximum value, and the temperature profile shows decreasing characteristics as the X ranges from 0 to 1. In other words, the temperature is maximum at the fin base and gradually reduces from there to the tip. Finally, there will be less temperature distribution at its tip. The porous fin has a lower thermal distribution than that of the solid fin owing to the occurrence of a large number of pores that allow fluid to flow through the holes. Consequently, a porous fin offers a greater rate of heat transmission than is achieved with a solid fin.

**Figure 4:**
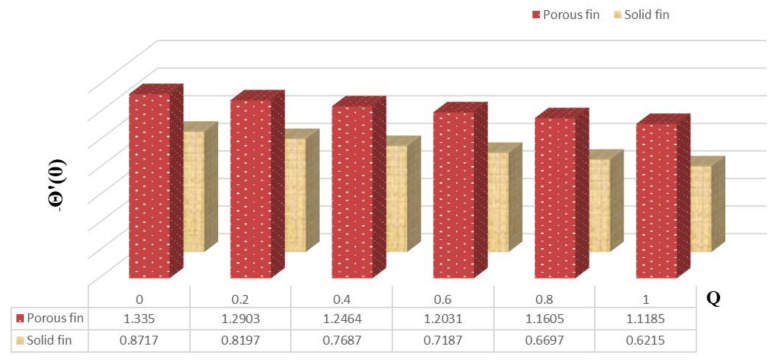
Heat flux of porous and solid fins.

Figure 4 delineates the variation in thermal flux for increased values of $Q_{\mathrm{int}}$ and also elucidates the comparison between solid and permeable fins. From this figure, one can notice that the thermal gradient decreases with enrichment in $Q_{\mathrm{int}}$ for both kinds of fins. Moreover, the heat flux is more for permeable fin compared to solid fin.

**Figure 5:**
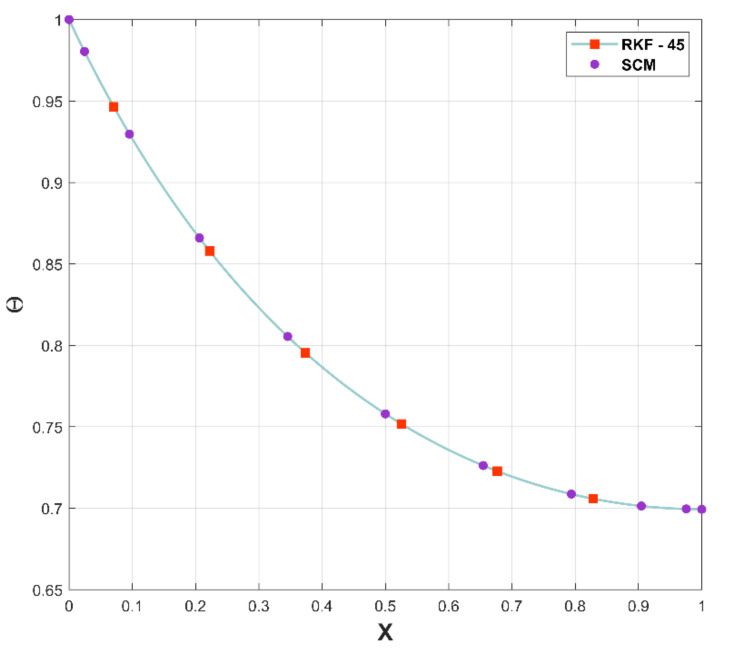
Comparison of the PR with RKF-45.

The SCM is a numerical method for more rapidly and effectively solving nonlinear differential equations. It has advantages over traditional finite difference approaches, including the ability to perform faster convergent approximations. The major purpose of plotting Figure 5 is to determine the accuracy of the considered numerical method, SCM, by comparing with RKF-45. As a result of the comparison of the two methods, an excellent pact has been recognized in the figure.

**Figure 6:**
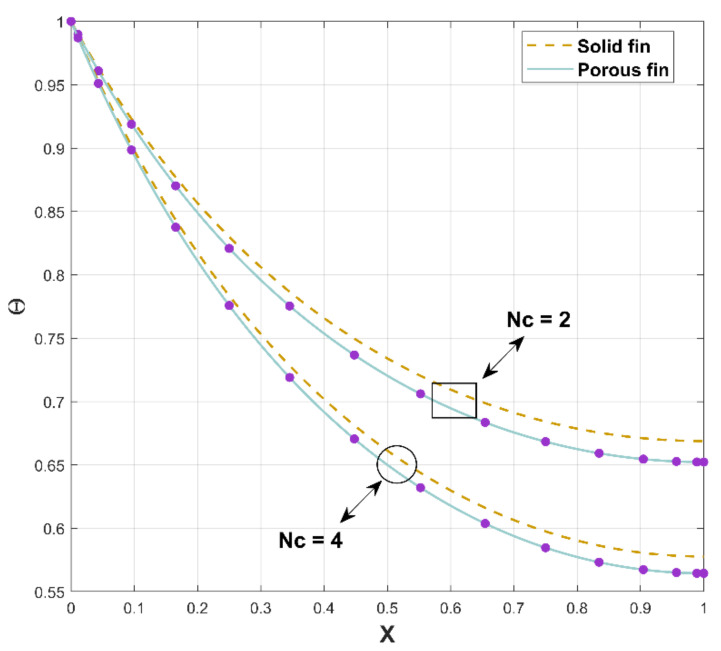
Thermal distribution through porous and solid fins.

The excellence of the proposed investigation can be spotted in Figure 6, which displays the nature of nondimensional temperature profile Θ as a function of Nc for solid and porous fins. The thermal profile of porous fin is reflected by solid thick lines in the figure, whereas dotted lines represent solid fin. For an increase in Nc, the thermal dispersion through both fins diminishes along the longitudinal direction. However, a solid fin has a higher thermal profile than that of a porous fin, as evidenced by the figure. As a result, heat transfer in dovetail porous fin is higher than that in solid fin.

**Figure 7:**
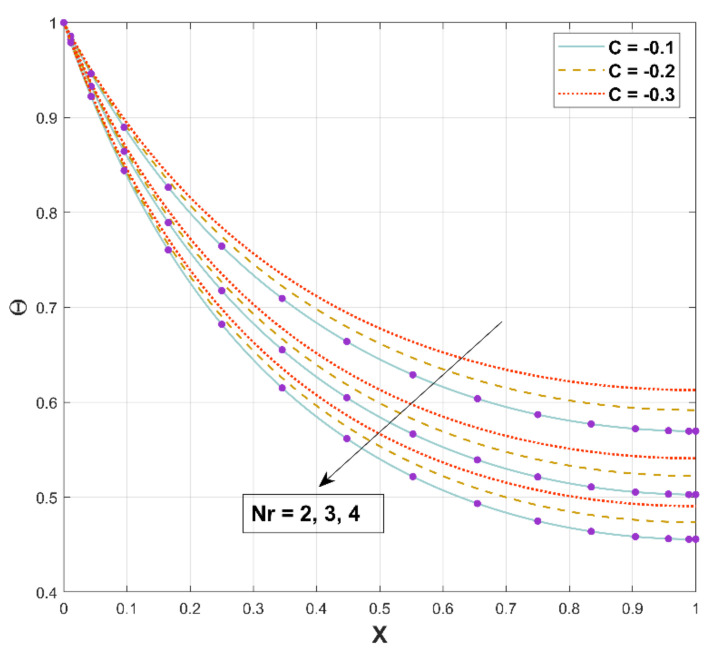
Upshot of Nr on Θ.

The deviation of the thermal field for the different coefficients of Nr has been embodied in Figure 7. This figure reflects that the temperature field diminishes with corresponding augmented values of Nr. The enhanced radiation results in effective heat dissipation from the fin surface to the adjacent fluid, as implied by the steadily declining temperature in DF. As an upshot, heat energy transmission induced by radiation proliferates the rate of heat transfer from DF.

**Figure 8:**
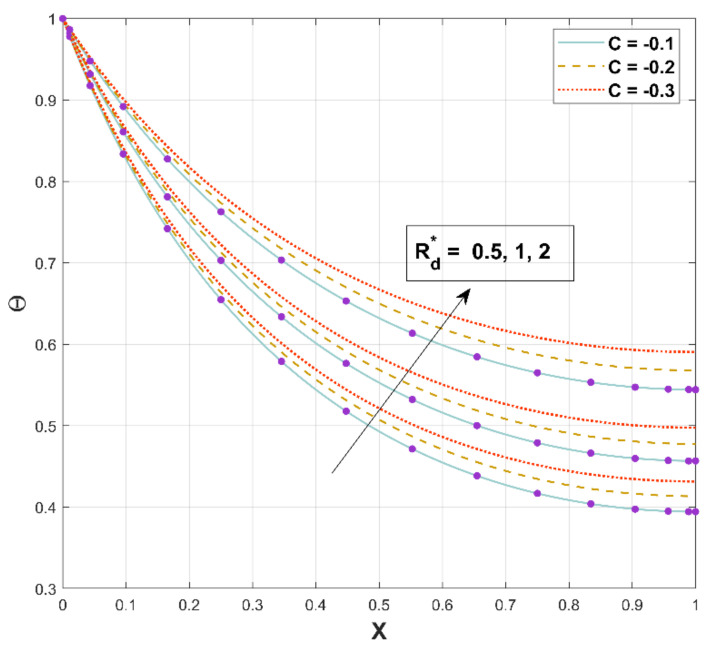
Upshot of $R_{d}^{*}$ on Θ.

Figure 8 characterizes the behavioral patterns of Θ for various $R_{d}^{*}$. Higher values of $R_{d}^{*}$ improve Θ, as can be seen in this illustration. The temperature in the fin increases as energy flux from heat dispersion and radiation is enhanced.

**Figure 9:**
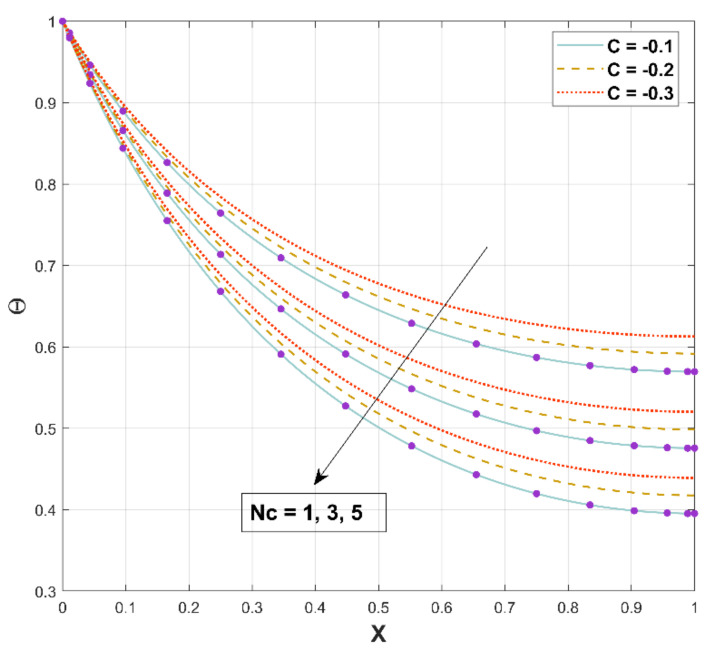
Upshot of Nc on Θ.

Surface convective heat transmission is involved in the rate of heat transmission and is indicated by the parameter Nc, which has the correlation between the convective and conductive processes. The influence of this parameter on the thermal field is indicated in Figure 9. According to this figure, improving this ratio lowers the temperature distribution. As the convection effect improves, more heat from the fin surface is transferred to the surroundings. The temperature consequently drops as the rate of heat transfer intensifies.

**Figure 10:**
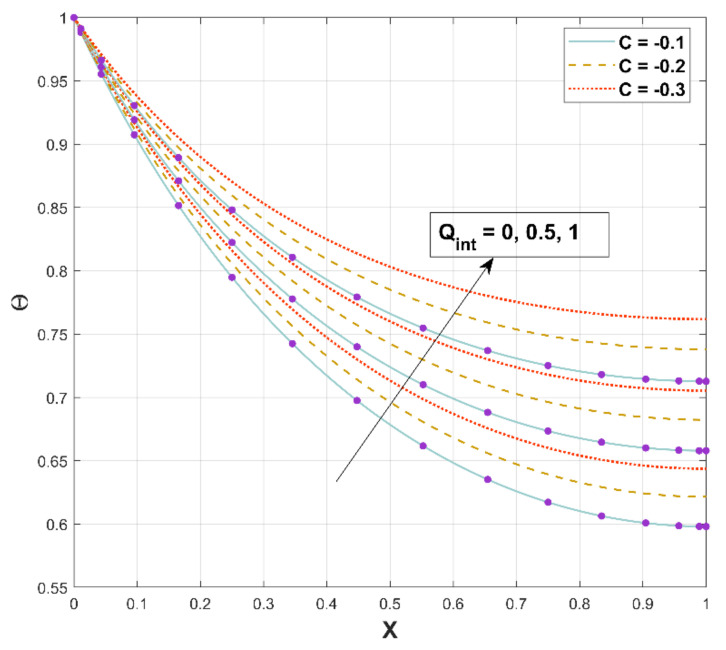
Upshot of $Q_{\mathrm{int}}$ on Θ.

Figure 10 is illustrated to exhibit the impact of $Q_{\mathrm{int}}$ on the thermal distribution along the longitudinal axis. Θ shows the upsurge behavior for increased estimations of the heat generation parameter. An upsurge in $Q_{\mathrm{int}}$ accelerates the internal heat generation (temperature-dependent), as conveyed in Equation (15). Advanced internal heat generation results in elevated Θ since a greater quantity of heat must be dispersed by the fin to its surroundings.

**Figure 11:**
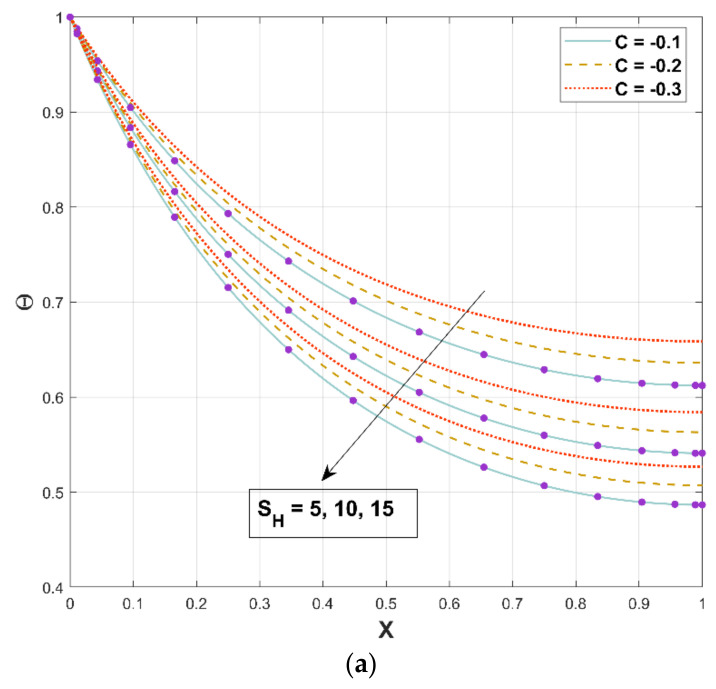

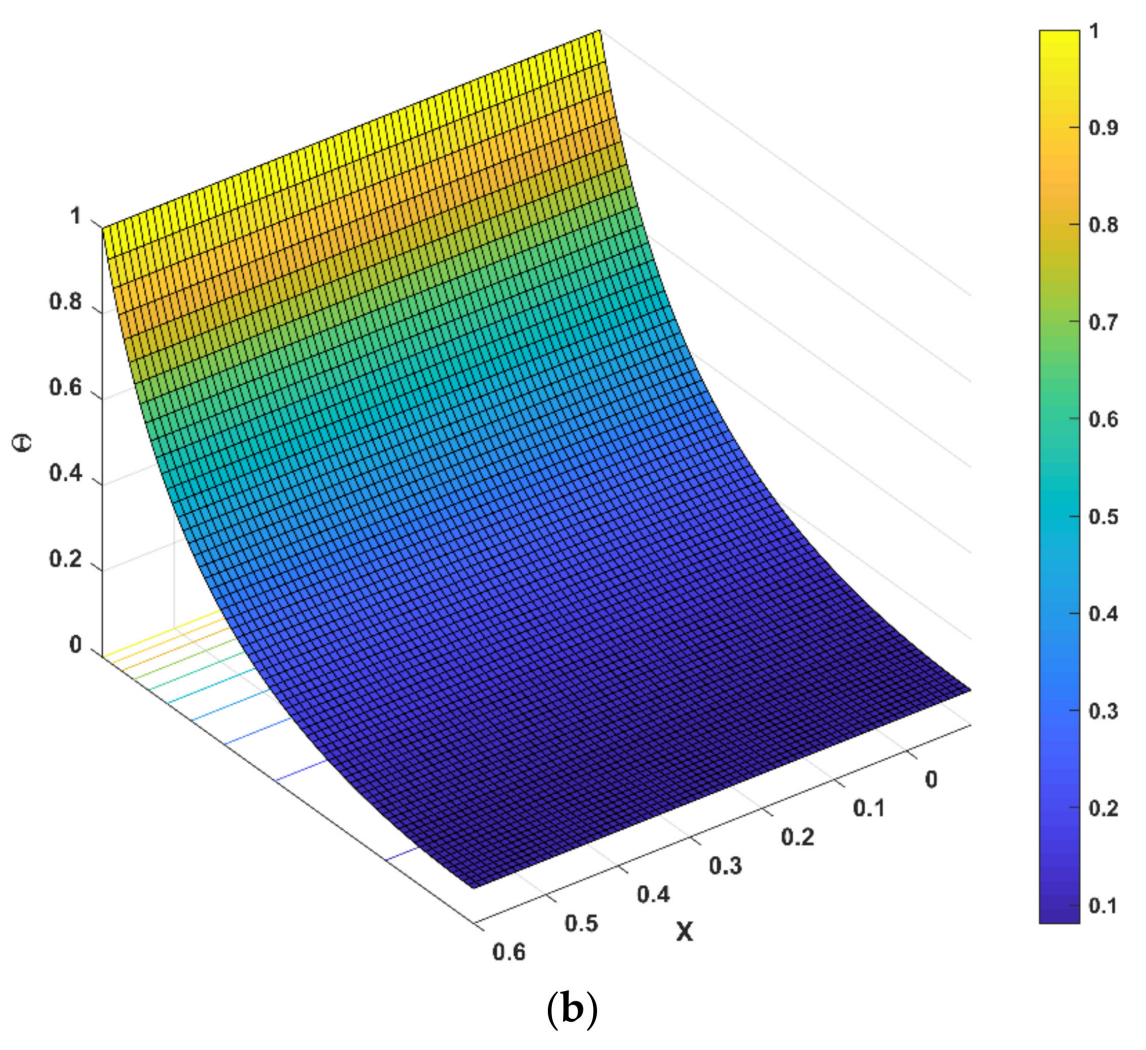
(**a**,**b**). Upshot of $S_{H}$ on Θ.

Figure 11a,b represents the relationship between the thermal field and the porosity parameter $S_{H}$. When this parameter is increased, the temperature distribution is reduced, resulting in a higher heat transfer rate, as displayed in Figure 11a. When steady-state heat transmission via a fin with porous media is examined under the impacts of natural convection and radiation, it is discovered that porous materials significantly improve heat transfer. Since a permeable fin is composed of pores and solid, heat interaction from the solid part of the porous fin is caused by direct exposure to the environment. The decreasing trend in fin temperature triggered by an increment in the porosity factor is based on the fact that as the porosity parameter improves, so does the porousness of the permeable fin, and thus the capacity of the working liquid to penetrate through the fin pores augments. Additionally, as the buoyancy force increases, more heat is dissipated, which accelerates the fin’s ability to transfer heat. In addition, a three-dimensional illustration is provided in Figure 11b for representing the nature of the thermal field for a specific value of the porous parameter. The energy dissipation rate lowers as the permeability improves, and the porous fin’s efficient thermal conductivity significantly reduces as the solid material is removed. Radiation and convection, on the other hand, improve the rate of heat removal. This variable symbolizes the temperature ratio, and an increase in this factor causes an increment in heat flux.

**Figure 12:**
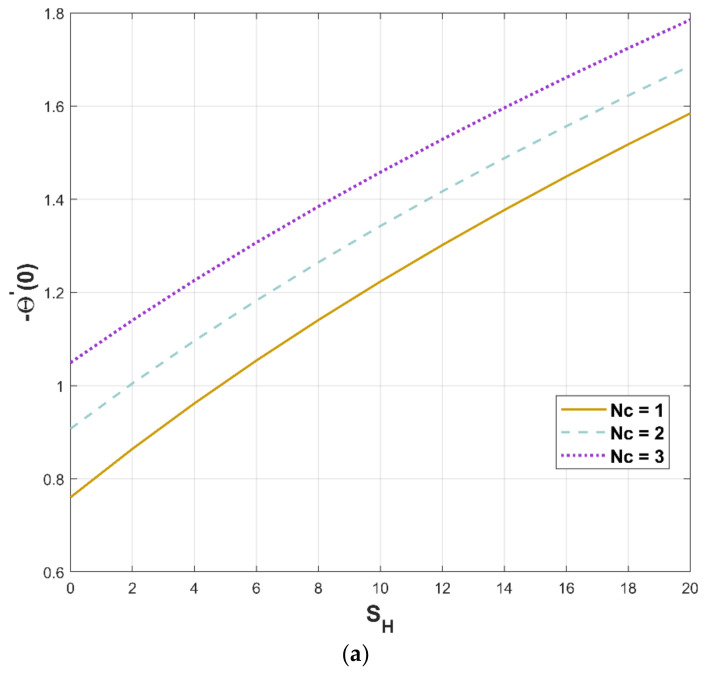

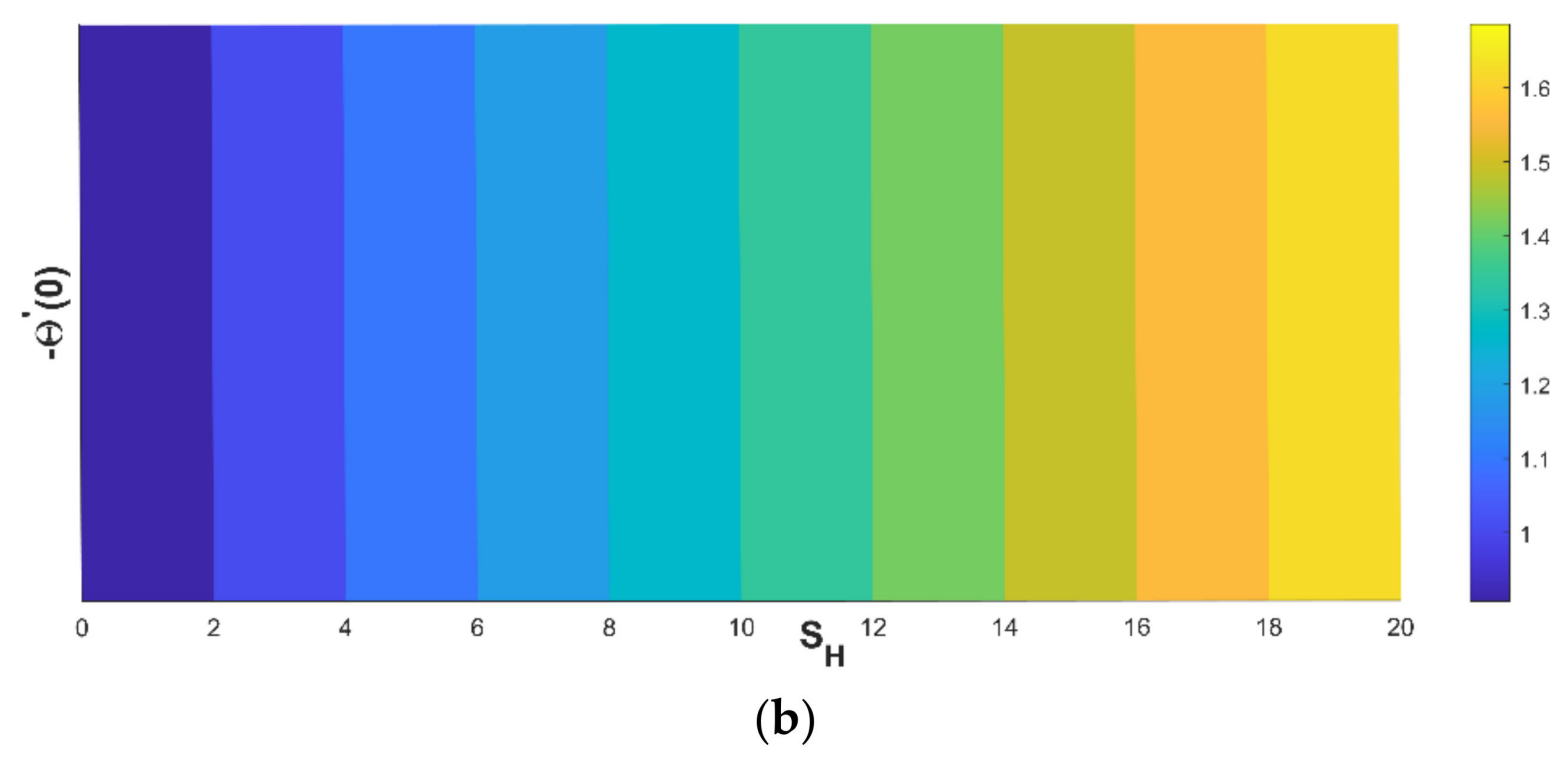
(**a**,**b**). Upshot of Nc and $S_{H}$ on $-\Theta^{\prime }\left(0\right)$.

Figure 12a,b illustrates the significant attributes of heat flux versus the convection–conduction parameter and the porosity parameter, respectively. According to the perceived notion, heat flux significantly augments when the convection–conduction parameter value is increased with respect to the porosity factor (Figure 12a), and an increment in thermal flux is caused by an increase in porosity factor (Figure 12b).

**Figure 13:**
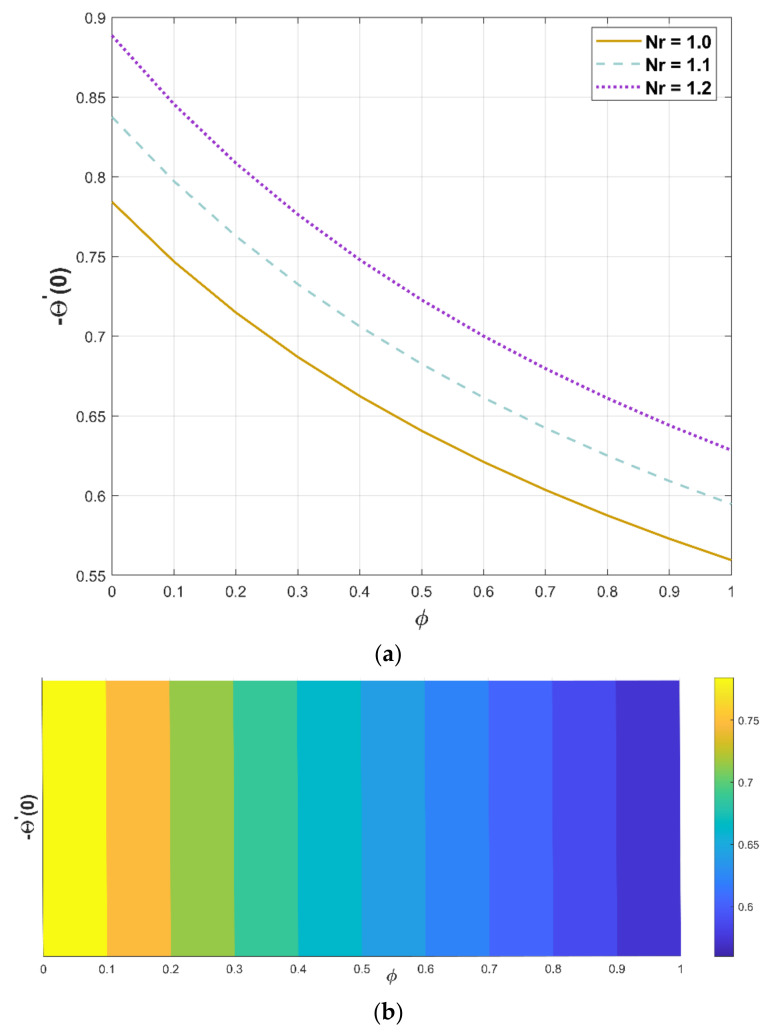
(**a**,**b**). Upshot of Nr and ϕ on $-\Theta^{\prime }\left(0\right)$.

Modifications in the quantity of Nr and porous factor have a consequence on the thermal flux, as revealed in Figure 13a,b. Figure 13a portrays the enriching behavior of heat flux as Nr increases against the porous factor. The heat flux decreases as a component of the porous factor, which is evidenced in Figure 13b.

**Figure 14:**
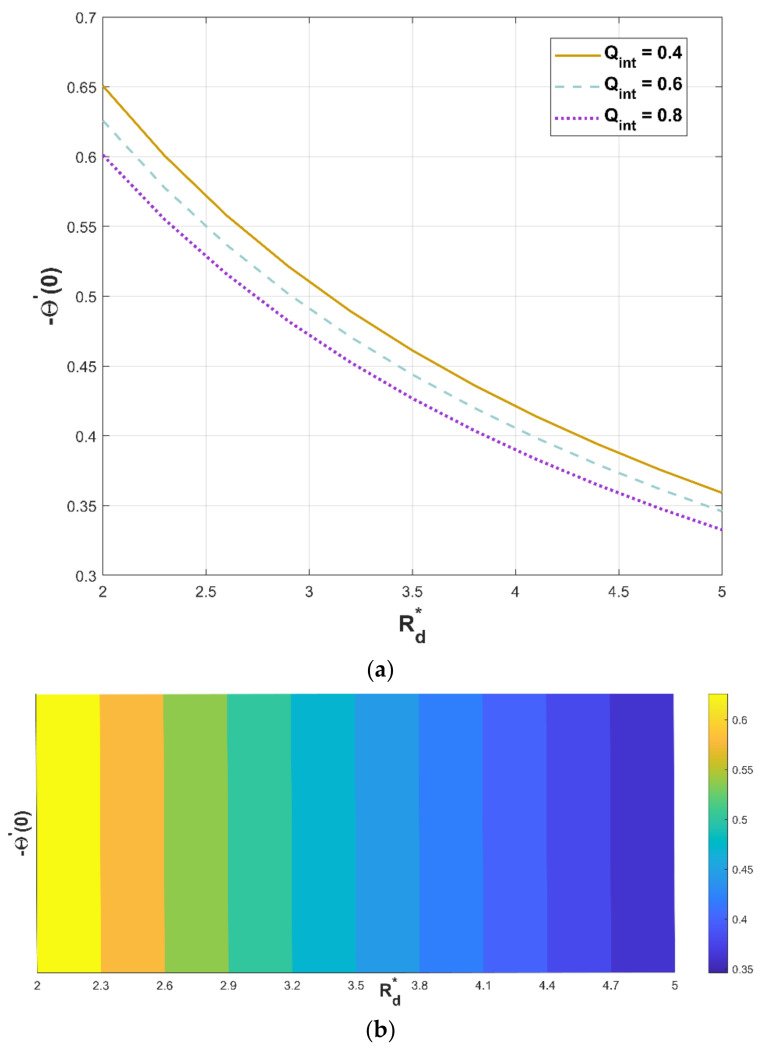
(**a**,**b**). Upshot of $Q_{\mathrm{int}}$ and $R_{d}^{*}$ on $-\Theta^{\prime }\left(0\right)$.

For a varying radiation–conduction parameter, Figure 14a,b portray the performance of the heat generation parameter on heat flux. As per Figure 14a, elevating values of $Q_{\mathrm{int}}$ significantly decreases the temperature flux against $R_{d}^{*}$. Greater values of $R_{d}^{*}$ implicate stronger radiative heat transfer and lead to an increase in the temperature profile, which causes less heat flux (Figure 14b).

For lower variability in $R_{d}^{*}$, it is found that the heat flux enhances. The fundamental notion for using permeable fin is to enhance the efficacious surface area through which heat is convected to the nearby liquid. Even though the effectual thermal conductivity of the permeable fin weakens due to the discharge of solid substance, this decline is balanced by an expansion in surface area. As a consequence, increased porosities significantly augment the heat transfer rate. As predicted, improving $R_{d}^{*}$ augments radiation heat exchange through the porous fin.

## 5. Conclusions

This analysis delves into the temperature behavior and heat transference of a permeable dovetail fin with internal heat generation. The nonlinear differential equation of a conducting–convecting–radiating dovetail fin with temperature-dependent thermal parameters is solved efficiently using SCM. The following are the major findings of the proposed study:

- SCM has an excellent agreement with the findings of available research, and to achieve precise results, only a few collocation points are required; thus, the technique is very effective in computation. This high level of accuracy suggests that the SCM is a viable alternative to other methods for solving fin problems with high nonlinearities.
- The thermal distribution through DF declines monotonically from the fin base to its tip, and the heat transfer rate in a porous fin is higher when compared to a nonporous fin.
- The temperature of the DF upsurges as the heat-generating number rises, which result in reduced heat flux.
- An increase in magnitude of the porosity parameter causes lower thermal dispensation and higher thermal flux in the fin.
- As the scale of the radiation number and convective–conductive parameter improves, the thermal distribution becomes lower and heat flux enhances through the dovetail fin.
- The increased magnitude of the temperature ratio parameter causes a decrement in the temperature distribution of the fin.
- The increment in the radiative–conductive variable leads to the upsurge of thermal dispersion and lessening of heat flux.

In the future, the numerical method can be extended to solve various fin models with more complex nonlinear differential equations. In addition, the current study can be extended to a transient thermal analysis of the dovetail fin under various physical mechanisms.

## Figures and Tables

**Table 2:** Numerical values of $\Theta \left(X\right)$ (at X=0.5) and $-\Theta^{\prime }\left(0\right)$ for different parameters when C=−0.3, n=0, $Q_{\mathrm{int}}=0.8$, and γ=0.1.

ϕ	$k_{r}$	Nc	Nr	Nt	$S_{H}$	$R_{d}^{*}$	$\Theta \left(X\right)$	$-\Theta^{\prime }\left(0\right)$
0							0.66972	0.32422
0.1							0.66394	0.33463
	0						0.66264	0.33536
	0.3						0.66652	0.33318
		2					0.66394	0.33463
		5					0.5071	0.47356
			2				0.59196	0.3729
			4				0.50587	0.40026
				0.1			0.72743	0.28435
				0.3			0.66394	0.33463
					0		0.68813	0.31494
					7		0.65494	0.34165
						0.5	0.67332	0.32714
						0.7	0.68322	0.32122

**Table 3:** Validation of present work with existing literature by taking parameters values as $C=Nc=n=Q_{\mathrm{int}}=\gamma =R_{d}^{*}=0$, ϕ=1, and $k_{r}=1$.

$S_{H}$	Nr	Nt	$-\Theta^{\prime }\left(0\right)$	
			Gorila and Bakier [46]	Present Result
1	0.1	0.01	0.68610	0.68613
		0.1	0.70210	0.70210
		0.5	0.83890	0.83892
	10	0.01	2.1763	2.1765
		0.1	2.6413	2.6415
		0.5	5.4420	5.4423
10	0.1	0.01	2.5616	2.5616
		0.1	2.5660	2.5660
		0.5	2.6100	2.6100
	10	0.01	3.2791	3.2792
		0.1	3.6059	3.6060
		0.5	5.9690	5.9693

## Data Availability

All relevant data are within the paper.

## Nomenclature

ν	Kinematic viscosity $\left(m^{2}\;s^{-1}\right)$	γ	Heat generation parameter (dimensionless)
g	Acceleration due to gravity $\left(m\;s^{-2}\right)$	$t_{b}$	Fin’s thickness $\left(m\right)$
C	Fin taper ratio(dimensionless)	T	Temperature $\left(K\right)$
q	Heat transfer rate $\left(W\right)$	t(x)	Fin’s semithickness $\left(m\right)$
w	Fin’s width $\left(m\right)$	Θ	Nondimensional temperature
$h^{*}$	Convective heat transfer coefficient $\left(W\;m^{-2}\;K^{-1}\right)$	$\upsilon_{w}\left(x\right)$	Average velocity of the liquid passing through the fin $\left(m\;s^{-1}\right)$
$q_{\infty }^{*}$	Heat generation at ambient temperature $\left(W\;m^{-3}\right)$	$c_{p}$	Specific heat $\left(J\;kg^{-1}\;K^{-1}\right)$
Nt	Temperature ratio parameter (dimensionless)	Nc	Convection–conduction parameter (dimensionless)
σ	Stefan–Boltzmann constant $\left(W\;m^{-2}\;K^{-4}\right)$	k	Thermal conductivity $\left(W\;m^{-1}\;K^{-1}\right)$
Nr	Radiation number (dimensionless)	$q_{\mathrm{int}}^{*}$	Heat generation $\left(W\;m^{-3}\right)$
$\varepsilon^{*}$	Emissivity	$S_{H}$	Porosity parameter (dimensionless)
L	Fin’s length $\left(m\right)$	$\beta_{R}^{*}$	Rosseland extinction coefficient $\left(m^{-1}\right)$
ρ	Density $\left(kg\;m^{-3}\right)$	$Q_{\mathrm{int}}$	Heat generation number (dimensionless)
x	Fin axial coordinate $\left(m\right)$	β	Thermal expansion coefficient $\left(K^{-1}\right)$
X	Length (dimensionless)	**Subscripts**	
ϕ	Porosity	∞	Ambient
$\dot{m}$	Mass flow rate $\left(kg\;s^{-1}\right)$	b	Base
$R_{d}^{*}$	Radiation–conduction parameter (dimensionless)	f	Fluid
$A_{cr}$	Cross-sectional area $\left(m^{2}\right)$	s	Solid
K	Permeability $\left(m^{2}\right)$	eff	Effective properties
